# “I’m Proud to be a Little Bit Different”: The Effects of Autistic Individuals’ Perceptions of Autism and Autism Social Identity on Their Collective Self-esteem

**DOI:** 10.1007/s10803-020-04575-4

**Published:** 2020-06-30

**Authors:** Rosalind Cooper, Kate Cooper, Ailsa J. Russell, Laura G. E. Smith

**Affiliations:** Department of Psychology, University of Bath, Claverton Down, Bath, BA2 7AY UK

**Keywords:** Autism spectrum disorders, Social identity, Self-esteem, Autism community, Autism strengths

## Abstract

This study aimed to identify the attributes that autistic people perceive as positively and negatively impacting on their identity and wellbeing. In Study 1, we recruited 140 autistic participants for an online survey. Participants completed autism social identification and collective self-esteem measures and listed attributes they associated with autism. In Study 2, we conducted focus groups with 15 autistic people to explore how positively they perceived the attributes of autism. Participants then discussed the autism attributes in relation to their own experiences and identity. We found a positive relationship between the number of positive attributes participants associated with autism, and their collective self-esteem, to the extent that they identified with other autistic people.

Autism is defined in the DSM-5 as a disorder, and historically clinicians and researchers have focused on ‘deficits’ associated with the condition. Yet, increasingly autistic people, clinicians, and researchers are focusing on and promoting the many strengths associated with autism. Previous research has demonstrated that this is likely to have positive outcomes; autistic people who identify with other autistic people and see the autistic community positively have improved mental health compared to other autistic people (Cooper et al. 2017). In the current research, we aimed to build on these previous findings to explore the specific attributes that autistic people associate with their condition and the extent to which they perceive these attributes to be positive. Second, we investigated the relationship between autism social identification and collective self-esteem and the number of positive or negative attributes autistic people link to this identity.

Autism is a neurodevelopmental condition that affects around 1.1% of people in the UK (Brugha et al. 2012). The Diagnostic and Statistical Manual of Mental Disorders (5th ed.; DSM-5 and American Psychiatric Association 2013) describes Autism Spectrum Disorder as characterised by difficulties with social interaction and communication, and a pattern of restricted repetitive and stereotyped behaviour. Research has contributed a broader understanding of the heterogeneity in the phenotype by highlighting neurocognitive differences such as executive function (Demetriou et al. 2018) and theory of mind (e.g. Jones et al. 2018).

Societal attitudes towards autism are beginning to shift from a focus on the challenges and difficulties faced by autistic individuals towards an acceptance of difference and an acknowledgement of strengths associated with the condition (Kenny et al. 2016). This is in the context of the disability rights movement and proliferation of online support communities for autistic people (Brownlow and O’Dell 2006). In Kenny et al.’s (2016) study, which surveyed 3470 members of the UK autism community, autistic individuals, family members and professionals expressed discomfort with using the terms ‘disability’ and ‘disorder’, preferring to use terms such as ‘difference’ and highlighting talents associated with the condition. While it is important to acknowledge strengths associated with autism, there are challenges implicit in the characteristics of autism, as individuals inhabit a social world which is set up for those without autism. Den Houting (2019) argues that it is essential to acknowledge these difficulties faced by autistic individuals, including those with intellectual disability, as part of the neurodiversity movement.

Much of the debate on terminology used to describe autism arises from differences in whether individuals feel that autism is a core part of their identity, or whether they prefer to distance themselves from the diagnosis (Cooper et al. 2017; Kenny et al. 2016). Individuals who believe that autism is inseparable from their identity are more likely to prefer ‘autism-first’ terminology, i.e., they refer to ‘autistic people’ as we do in this paper. Others prefer ‘person-first’ terminology, implying that autism is not the primary way to identify an individual, and so would say ‘person with autism’. Gernsbacher (2017) argues that person-first language in fact increases stigma as it tends to be used more for children and those with more stigmatised disabilities, and that identity-first language is destigmatising and encourages positive identification with disability which is associated with positive outcomes.

Social identity theory (SIT; Tajfel and Turner 1979) provides a useful lens through which to understand the debates around autism terminology. SIT posits that as humans we have a propensity to form ourselves into social groups and to define ourselves by these group memberships. This leads to behavioural patterns such as favouring members of the in-group over those who do not belong to the group. It also results in a sense of well-being and self-esteem as individuals derive positive feelings of affiliation towards others in the group and a sense of pride in group membership (Haslam et al. 2012). The use of autism-first terminology by autistic people suggests a strong sense of group membership and, potentially, positive feelings towards belonging to the group. Non-autistic people may also use this terminology in solidarity with the autism community.

Deriving a positive sense of self from autism is complicated by the fact that autism is stigmatised in society. This presents challenges to deriving a sense of self-worth from group membership. Many autistic people experience high levels of stigma, facing bullying (Cappadocia et al. 2012) and social isolation (Billstedt et al. 2011). Membership of a stigmatised group can result in negative outcomes for emotional wellbeing (Crocker and Major 1989), such as increases in anxiety and depression (Katz et al. 2002). Indeed, Botha and Frost (2020) demonstrate that experiences of discrimination, internalised stigma and concealment of identity in autistic people were associated with poorer mental health. However, a positive sense of self-worth can still be derived from group membership in a stigmatised group, if individuals develop *collective self-esteem*, or positive feelings or acceptance of the shared group identity and the strengths and talents of the group (Hurlbutt and Chalmers 2002; Cooper et al. 2017; Cage et al. 2018).

Autistic individuals who feel a strong sense of affiliation with other autistic people, and who have positive views about the autistic community (or in social identity terms, they have high *social identification* as a member of the autistic community), have improved individual self-esteem and psychological well-being as manifested by lower depression and anxiety scores (Cooper et al. 2017). This suggests that social identity processes as described above are a useful framework for understanding identity and well-being in the autistic community, and perhaps a useful tool for interventions to improve wellbeing. Indeed, the extent to which an autistic person accepts their autism identity, and feels society accepts their autism, has been found to be negatively associated with depression scores in autistic adults (Cage et al. 2018). However, a study with 24 autistic adolescents found that autistic individuals who aligned themselves to non-autistic culture generated more positive self-statements than those who did not align themselves with either autistic or non-autistic culture (Cresswell and Cage 2019). Mixed findings about how positively autism identity is perceived are prevalent in qualitative research in the field, with some participants emphasising strengths, others emphasizing challenges and many with a balanced perspective (Macleod et al. 2013; Mogensen and Mason 2015; Cage et al. 2016). Indeed, a key theme arising from a qualitative investigation of autistic strengths with 24 autistic adults was “false dichotomies” (Russell et al. 2019). This theme referred to how autistic attributes which at times were strengths could at other times represent challenges. These studies show that there are both strengths and challenges associated with autism, and the mixed findings are likely to represent the mixed views of many autistic individuals towards autism, and whether this is perceived as an identity to embrace or one from which to distance oneself.

In sum, there is evidence that some autistic people prefer to see autism as integral to their identity, and that having a positive sense of autism identity has benefits for mental health. However, there is also evidence that autism is seen negatively by some individuals. We therefore do not have an adequate understanding of how autistic people themselves perceive autistic attributes, and whether this extends beyond the traits described in diagnostic manuals. A systematic investigation is needed of the attributes that autistic people associate with autism, their lived experience of these attributes, and how negatively or positively these attributes are perceived to be. This would help to improve understanding of how autistic people themselves conceptualise autism, and how this affects how they feel about themselves, which has implications for mental wellbeing in this group. Accordingly, this study aims to explore the attributes of autism, the lived experience of these attributes, and how positively or negatively these features are perceived by the autistic community. We hypothesised that participants who listed more attributes that other autistic people view as positive, would have higher identification with autism and, in turn, collective self-esteem.

## The Present Research

This research included two studies and an analysis that integrated the results of the two studies. Study 1 was an online survey completed by autistic adults to generate autism attributes and measure autism social identification and collective self-esteem. In study 2, autistic people rated the autism attributes collected in Study 1 according to how positive these were perceived and discussed their personal experiences of these attributes. Then, in an integrative analysis, we applied the positive and negative valence ratings to the Study 1 data to investigate the associations between attribute valence, autism social identification, and collective self-esteem.

### Ethics

Ethical approval was sought and granted by the University Ethics Committee (16-024). All study materials were reviewed by autistic people prior to the study commencing. Participants were informed of their right to withdraw, gave fully-informed consent, and all data was anonymised.

## Study 1

### Participants

Participants were recruited via online forums, the National Autistic Society (NAS) research network, and social media advertisements. Autistic participants over the age of 16 years were invited to complete the study. This was defined to include Asperger’s Syndrome, High Functioning Autism, Autism, Atypical Autism, Pervasive Developmental Disorder (PDD) and PDD-Not otherwise specified. In total, 140 participants completed the online survey with a mean age of 36 years (*SD* = 15, Range 16–70). Of the participants, 123 (88%) were White, 9 (6%) were from mixed or multiple ethnic backgrounds, 2 were Asian (1%), 3 were Black (1%) and 3 were from other ethnic backgrounds (2%). Seventy-one participants (51%) were female, 51 (36%) were male and 18 (13%) preferred not to say. Seventy-five percent reported having received a formal diagnosis of autism, at a mean age of 29.19 years (*SD* = 14.89). In the diagnosed group (n = 105), 38% were male, 53% female and 8% preferred not to say, and the average age was 34 years (SD = 14.57). In the self-identified group (n = 35), 31% were male, 43% were female and 14% preferred not to say, and the average age was 40 years (SD = 14.46).

### Procedure and Measures

Participants completed a series of measures (see below). Following these, basic demographic information was collected, including age, ethnicity, gender, and information regarding autism diagnosis.

#### Autism Social Identification

We measured the extent to which participants felt that autism contributed to their self-definition, or identity, using the autism social identification measure (Cooper et al. 2017). This was adapted from the Multi-dimensional Scale of Social Identification (Leach et al. 2008), which has been shown to be reliable and valid across different social identities and had good internal consistency in this study (α = .91). It contained 14 items scored from 1 (strongly disagree) to 7 (strongly agree) which captured two dimensions—self-investment and self-definition. Example items include, “I often think about the fact I have autism” and “people with autism are very similar to each other”. A higher score is indicative of a stronger identification with an autism social identity, i.e., higher affective and cognitive commitment to the group, self-stereotyping, and sense of in-group homogeneity. Possible scores range from 1 to 7.

#### Collective Self-esteem

We used a measure of collective self-esteem to capture the extent to which being in a group with other autistic people positively contributed to participants’ self-esteem. This measure was adapted by Cooper et al. (2017) for use with autistic people from the Private and Public Collective Self-esteem Subscales (Luhtanen and Crocker 1992), which has been assessed as reliable and valid (α = .67). The measure includes eight items rated on a seven-point Likert scale from strongly disagree (1) to strongly agree (7) with higher scores indicating greater collective self-esteem.

#### Autism Attributes

Participants were asked to “list up to 5 words or phrases that you think are to do with autism”. Participants responded in a free text box. The autism attributes were grouped into categories of attributes via inductive content analysis (Hsieh and Shannon 2005). The first coder grouped responses into themes of words or phrases that were the same or referenced semantically related constructs e.g. “predictability is important” and “dislikes change”. This grouping was validated independently by another researcher. Discrepancies were discussed and re-categorised if necessary. If the original raters could not reach agreement, a third coder was consulted, and the consensus code was recorded. Each group was ascribed an overall attribute name using the same process with a second researcher. The top ten most frequently reported attributes were recorded and discussed in the Study 2 focus groups (see Table 1). Participants’ responses to the autism identification and collective self-esteem measures were reserved for use in the integrative analyses (described below).

**Table 1:** Attributes of autism, study 1

Attribute	Attribute	Frequency, N (%)	Example words/phrases within attribute
1	Social skill difficulties	60 (43%)	Social awkwardness, difficulty relating to others
2	Loneliness	50 (36%)	Isolation, friendless, loner
3	Sensory issues	43 (31%)	Sensory difference, sensory overload, hypersensitive
4	Emotional difficulties	35 (25%)	Depression, struggle, meltdown
5	Difference	29 (21%)	Being different, different not less
6	Cognitive differences	29 (21%)	Different perspectives, processes input differently
7	Anxiety	29 (21%)	Anxious, panic, OCD
8	Communication issues	28 (20%)	Uncommunicative, communication problems
9	Gifted	24 (17%)	Smart, intelligence, talented
10	Unique	23 (16%)	Unique, nonconformist, eccentric
11	Special interests	22 (16%)	Having strong interests, intense interests
12	Caring	22 (16%)	Empathetic, supportive, kind
13	Bullied	20 (14%)	Oppressed minority, discrimination, being bullied
14	Routines	17 (12%)	Routine, order, predictability
15	Focused	15 (11%)	Hyper-focus, intense focus
16	Stimming	12 (9%)	Stimming (fidgeting/rocking), self-stimulating movements
17	Attention to detail	11 (8%)	Exceedingly detail oriented
18	Rational	10 (7%)	Logical ability, scientists, objective
19	Introversion	10 (7%)	Likes own company, quiet, introverted

## Results

In Table 1 we present the attributes generated by 10 or more participants. Quantitative findings relating to the autism identification and collective self-esteem measures are presented in the Integrative Analyses section below, and in Table 4. On average, participants reported 4.5 attributes. Forty-three attributes were mentioned by more than one participant, and there were 55 idiosyncratic attributes mentioned by individual participants that were not categorised. Examples of uncategorised attributes were, “freedom” and “trivia”. The attribute identified by most participants was “social skill difficulties”, with 43% of respondents listing this in one of their five open text-boxes. There was significant agreement on the top eight attributes, with more than 20% of the sample mentioning these attributes. These attributes span the core characteristics of autism as outlined in diagnostic manuals as well as reflect emotional difficulties and social experience of autistic people.

## Study 2

### Participants

We recruited focus group participants (*N* = 15) from a university transition programme for autistic students and a community social group for autistic adults. None of the participants in Study 2 had taken part in Study 1. The second study was advertised verbally at the university transition programme and at the community social groups, and focus groups took place during these activities to allow interested individuals to participate. There were four focus groups in total. Two focus groups had student participants (*n* = 10), with a mean age of 18.67 years (*SD* = 4.27), and mean age of autism diagnosis was at 14.56 years (*SD* = 7.33). Two focus groups included community participants (*n* = 5), and in these groups the average age was 35.5 years (*SD* = 20.37), with a mean age of autism diagnosis of 29.60 years (*SD* = 18.13).

Across both focus groups, nine (60%) participants were male, five (33%) were female, and one (7%) described their gender as ‘other’. Twelve participants (80%) were White British, two were White and Black Caribbean (13%) and one was Chinese (7%) All focus group participants had received a formal diagnosis of Autism Spectrum Disorder.

### Procedure

Focus groups were conducted over a single 60-min session. There was one facilitator per focus group. Participants were instructed that they would be presented with autism attributes as identified by a different group of autistic participants, and that they would be asked to rate how positively or negatively they thought of each attribute. They would then be given chance to discuss their rating, with no pressure to share their rating or contribute to the discussion if they preferred not to. The focus group participants were then presented with each of the top ten autism attributes in turn. Each individual then gave each item a score in writing from − 5 to + 5 relating to how positive they thought it to be (where − 5 = not positive at all and + 5 = extremely positive). A mean rating was calculated for each attribute.

Participants explained and discussed their ratings for each item, to allow us to gain a deeper understanding of how autistic participants interpreted the autism attributes in relation to their own experiences and identity, adding depth to the quantitative dataset collected in the Study 1 survey.

The focus group discussions were audio recorded and transcribed by the first author, with identifying details removed. We conducted a thematic analysis of the focus group transcripts (Braun and Clarke 2006) to discover lived experiences of autistic people of the autism attributes identified in Study 1. We used an inductive approach to ensure the themes identified were data driven, rather than based on prior theory (Boyatzis 1998). We conducted the analysis on the transcripts from all the focus group audio recordings to identify common themes across the entire data set. The procedure entailed line-by-line coding of the transcript, followed by looking for themes to group the codes, which were developed by constant referencing of the transcript, codes and themes and re-reading the dataset (Braun and Clarke 2006). The primary data analyser was a trainee clinical psychologist who was completing a doctorate within the university department that hosted the university transition program. She did not know any of the focus group participants personally or professionally from either the student or community group samples. The research was conducted from a critical realist perspective. This assumes that the data collected through the focus groups reflects the participants’ mental states, including intentions and meanings (Gorski 2013).

## Results

### Ratings of Valence of Autism Attributes

An average rating of each of the focus group participants’ scores was taken for each of the 10 autism attributes (see Table 2).

**Table 2:** Attribute ratings across focus groups

Attribute	Average ratings^a^					
	Group 1	Group 2	Group 3	Group 4	Overall	Classification^b^
Gifted	4	2.5	2.2	− 0.6	1.67	Positive
Unique	3.7	3	1.3	− 0.4	1.43	Positive
Difference in cognitions	3	0.5	0	1.8	1.27	Positive
Difference	− 0.7	− 1	0	0.6	0.2	Neutral
Sensory issues	− 0.3	− 3	− 2	− 1.6	− 1.67	Negative
Loneliness	1	− 2	− 2.8	− 2.3	− 1.77	Negative
Social skill difficulties	− 2.3	− 0.5	− 2.7	− 2.6	− 2.3	Negative
Communication issues	− 2	− 1	− 2.9	− 3	− 2.5	Negative
Emotional difficulties	− 2.3	− 3	− 2.1	− 2.8	− 2.5	Negative
Anxiety	− 1.7	− 4	− 3.6	− 4	− 3.4	Negative

^a^Groups one and two were the community focus groups, three and four were student groups

^b^We conducted a within-samples t-test on the overall mean for each attribute to check that they were significantly above or below the mid-point of the scale; all attributes were significantly different from the midpoint with the exception of ‘difference’ which was classified as ‘neutral’

### Thematic Analysis of Transcripts of Focus Group Discussions

We identified four overarching themes in the data analysis. See Table 3 for the overarching themes, sub-themes, and supporting quotes which were selected as best representing each theme.

**Table 3:** Overarching themes, subthemes and supporting quotes from student focus groups (n = 10) and community focus groups (n = 5)

Overarching theme	Subtheme	Example quote
Challenges due autism	Intrinsic difficulties	“I’ve had a few emotional difficulties from time to time … it just seems to be there’s always a hurdle that needs to be climbed, or mountain that needs to be jumped when you have Asperger’s or Autism”
	Rejection by others	“…all throughout my school I’ve been bullied or excluded in various ways … It’s still really bad, I still spend a lot of time alone, because of my social issues and other people not accepting me.”
	Lack of understanding from others	“People assume ‘oh, you’re autistic, you know what year Henry VIII died’—you know, stuff like that. So people put this level of, of veneer on you, or this level where you’re expected to deliver, and sometimes you can’t deliver”
Diversity and adaptation	Autism as a spectrum	“…the spectrum is quite varied, and quite—you know, you might be able to tick the box in some areas, but not in others”
	Effect of environment	“…it very much depends on the environment, because I know someone who is a scientist, who does really well, and contributes important things, but if he was in the wrong environment he would really really struggle.”
	Improvements with time	“But now I’ve kind of like matured a bit more, my confidence has gone up, it’s not really so much of a problem, being lonely now.”
	Diagnosis leads to positive change	“When I got diagnosed everything started to fall into place, and I started to look at myself, and how I can improve on the bad attributes, the loneliness and everything else.”
Navigating difference	Self-acceptance	“It is important to kind of understand yourself, and be well in yourself.”
	Resilience	“But also when I was younger, someone said—these older guys was in the toilets and they were like ‘oh, why can’t you be normal like everyone else?’ and I just turned around and said ‘I don’t want to be a clone’—and his mate laughed at him and said ‘he got you there’.”
Positive autism identity	Advantages of autism attributes	“I’ve lived with myself for long enough to know I have got lots of advantages that other people haven’t got.”
	Autism pride	“I’m proud to be a little bit different—I thrive from being a little bit different, it makes me unique to who I am.”

#### Challenges Due to Autism

This was the first over-arching theme, which referred to the difficulties participants experienced due to the features of their autism, and responses of others to their autism. The *intrinsic difficulties* subtheme captured challenges due to being autistic, including associated emotional difficulties such as anxiety, alongside as social and sensory challenges. Such challenges could lead to frustration and anger. Regarding *rejection by others,* participants, particularly in the student groups, reported receiving negative treatment from others due to the differences that autism brings. *Lack of understanding from others* referred to incidents where other people ascribed stereotypical views of autism to individuals, demonstrating a lack of understanding of the individual beyond their diagnosis.

#### Diversity and Adaptation

This was about how autism is not experienced in the same way by every individual, and that the features of autism are affected by context, whether environmental context, age of the individual, or time since autism diagnosis. The *autism as a spectrum* theme arose when participants highlighted that all humans are unique and experience challenges and have strengths, just like autistic people. The *effect of environment* subtheme captured how autistic attributes could be affected by the environment positively or negatively. External stimulation, sensory factors and social situations were common examples. The *improvements with time* subtheme arose because participants described several difficult aspects of autism as improving over time. Some participants explicitly linked these improvements to increased understanding and self-acceptance as they grew older, while others linked this to an increase in skills, e.g. time management. The final subtheme was *diagnosis leads to positive change*. Several participants discussed the positive impact of receiving a diagnosis of autism, as it led to increased understanding and support.

#### Navigating Difference

The overarching theme ‘navigating difference’ captured participants’ strategies for managing their autism, having an identity which differed from the norm and living in a world designed for non-autistic people. The *self-acceptance* subtheme referred to participants’ statements that developing an understanding of themselves and their autism was an important way to improve their wellbeing. The *resilience* subtheme highlighted participants’ tendency to find positive aspects of difficult situations, and to make adaptations when necessary.

#### Positive Autism Identity

This theme highlighted the positive sense of identity many autistic participants experienced. The first subtheme was about the *advantages of autism.* Examples of such benefits included creative thinking, thinking outside the box and not being constrained by social norms. Advantages were discussed to a greater extent in the community focus groups compared to the younger student groups. *Pride in difference* referred to participants’ views that their differences from non-autistic people were a positive part of their identity. For example, participants were proud that they did not follow the crowd and acted in a way that was true to themselves, which could lead to more progress and diversity of thinking within society.

## Integrative Analyses

In this analysis, we aimed to calculate how positively or negatively autistic participants conceptualised autism. We did this by using the focus group scores, which established whether the autistic participants in the study perceived various attributes of autism as positive or negative, on average. First, we calculated the average score for each attribute from the Study 2 focus groups. Each attribute was then rated as being either ‘positive’, ‘neutral’ or ‘negative’ (see Table 2). Attributes were labelled as positive if the Study 2 rating for the attribute was above 1. Attributes were labelled as neutral if the rating was between − 1 and 1. Attributes were labelled as negative if the rating was below − 1. We then went back to the Study 1 participants and identified the attributes that they had associated with autism, and whether these had been rated as positive, which was scored as 1, neutral, which was scored as 0, or negative, which was scored as − 1. We then calculated an average score for the five attributes identified by each Study 1 participant. This allowed us to calculate a ‘valence of autism attributes’ score, indicating how many positive or negative attributes Study 1 participants associated with autism (see Table 4), with a minimum score of − 5 and maximum score of 3.

**Table 4:** Descriptive statistics for participants formally diagnosed with autism (n = 105), those who self-identified autism (n = 35), and for the total sample (N = 140), Cronbach’s alpha, and partial correlations for all participants controlling for gender and age

Measure	Autism diagnosed		Autism self-identified		Total sample				
	*Mean*	*SD*	*Mean*	*SD*	*Mean*	*SD*	1	2	3
1. Autism social identity	4.26	1.12	4.48	0.97	4.32	1.08	(0.91)	.52**	.41**
2. Collective self-esteem	15.14	3.70	15.82	2.96	15.31	3.53		(0.67)	.24*
3. Valence of autism attributes	− 1.18	1.60	− 1.24	1.70	− 1.19	1.60			-

**p < .001, *p < .01

We conducted a partial correlation analysis to investigate the associations between autism social identification, collective self-esteem, and the valence of autism attributes score, controlling for gender and age. We controlled for these variables due to qualitative findings that younger participants were less likely to discuss advantages of autism compared to older participants, and for gender due to quantitative findings that it had an effect on autism identification. We also included all participants in this analysis as there were no significant differences between the groups (diagnosis versus no diagnosis) on each of these measures.^1^ When controlling for gender and age, we found a significant positive association between valence of autism attributes and autism social identification (*r* = .41, *p* < .001), and between valence of autism attributes and autism collective self-esteem (*r* = .24, *p* < .05). We also found a positive association between autism social identification and autism collective self-esteem (*r* = .52, *p* < .001).

**Fig. 1:**
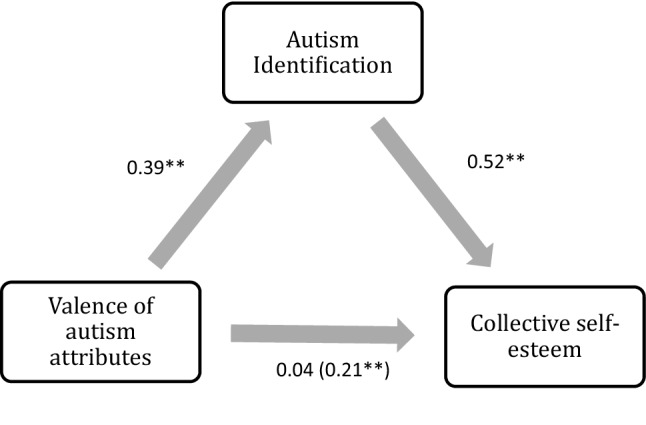
Standardised regression coefficients for the relationship between valence of autism attributes and collective self-esteem as mediated by autism identification. The standardised coefficient for the relationship between valence of autism attributes and collective self-esteem, controlling for autism identification, is shown in brackets. **p < .001

Using Hayes’ (2018) PROCESS macro (Model 4) with 5000 bootstrap samples, we conducted a mediation analysis to test the indirect relationship between valence of autism attributes and collective self-esteem through autism social identification, controlling for gender and age (see Table 5 and Fig. 1). There was no significant effect of age on autism identification, β = 0.04, *p* = .62, but there was a significant effect of gender, β = 0.36, *p* < .001. There was a significant positive relationship between valence of autism attributes and autism social identification, β = 0.39, *p* < .001. There was no significant effect of age on collective self-esteem, β = − 0.53, *p* = .36, nor gender on collective self-esteem, β = − 1.20, *p* = .06. There was a significant relationship between autism social identification and collective self-esteem, β = 0.52,* p* < .001. The direct effect between valence of autism attributes and collective self-esteem was non-significant, β = 0.04*, p* = .68. However, there was a significant indirect relationship between valence of autism attributes and collective self-esteem through autism identification β = 0.21 (95% Bootstrapped Confidence Intervals = 0.11, 0.31).

**Table 5:** Analysis of direct and indirect paths controlling for gender and age (*n* = 133)

Path	β	*B*	95% confidence intervals for *B*
(a) Valence of autism attributes → autism identification	0.39	0.27	0.17–0.37
(b) Autism identification → collective self-esteem	0.52	3.40	2.28–4.52
(c) Valence of autism attributes → collective self-esteem	0.04	0.15	− 0.57–0.88
(c’) Valence of autism attributes → autism identification → collective self-esteem	0.21	0.91	0.47–1.44

## General Discussion

We aimed to investigate the attributes which the autistic community associate with autism, and to find out their lived experience of these attributes. We further aimed to test the hypothesis that associating more positive attributes with autism (as rated by focus-group members) would be positively associated with individual identification with autism and, in turn, collective self-esteem. We identified a broad range of attributes of autism and focus group participants disclosed pride in their autism and described experiencing their autism differently over time and dependent on context, as well as experiencing challenges relating to autism. In support of our hypothesis, a mediation analysis found that autistic participants who associated positive attributes with autism had improved collective self-esteem, to the extent that they had a strong affiliation with their autism identity.

### Theoretical Implications

While the core diagnostic features were included in the autism attributes, broader issues, particularly emotional difficulties such as anxiety and depression were put forward by many participants as key features of autism. This is unsurprising in the light of reports of the high rates of conditions described as ‘co-occurring’ with autism. For example, the pooled estimate of the lifetime prevalence of anxiety in the adult autism population following meta-analysis has been found to be 42% (Hollocks et al. 2019). Our findings imply that autistic people may view emotional difficulties as integral to autism rather than as ‘co-occurring’ conditions with distinct aetiology. Alternatively, our findings may reflect emotional and psychological processes which are transdiagnostic and shared across a range of neurodevelopmental conditions. Moreover, some of the attributes focused on strengths such as “gifted” and “unique”, in line with the increasing focus on positive aspects of the autism diagnosis and identity (Kenny et al. 2016; Russell et al. 2019). It is noteworthy that a large proportion of attributes listed were idiosyncratic. i.e. mentioned by individual participants in Study 1. This highlights the diversity of perceptions of autism in the autistic community.

In terms of the experience of the attributes listed, greater numbers of these were viewed negatively by autistic people. Furthermore, the magnitude of the negative ratings was greater than those of the positively rated attributes. This suggests that the attributes of autism were predominantly perceived as negative by autistic people, which, according to social identity theory, has negative implications for psychological wellbeing (Haslam et al. 2012). However, the positively rated attributes contribute to an understanding of the components of collective self-esteem in autism, which could be helpful in modifying the impact of a negative social identity. The finding that participants rated some attributes as positive, others as neutral and negative, fits with previous qualitative research which has found a range of opinions in the autism community about having an autism identity (Macleod et al. 2013; Mogensen and Mason 2015; Cage et al. 2016). We found a similar range of opinions. In particular, the younger participants in the student focus group spent more time discussing challenges than adults in the community group, which is perhaps unsurprising in the context of bullying at school (e.g. Cappadocia et al. 2012), and being bullied and discriminated against being the 12th most listed attribute of autism.

We found that study 1 participants who listed more positive attributes of autism (as rated by study 2 participants) felt more positive about autism group membership to the extent that they identified with other autistic people. This supports the theory that individuals belonging to a stigmatised social group can preserve psychological well-being and self-esteem by developing a positive sense of their identity and strengthening connections with other autistic people (Blanz et al. 1998). This supports findings from a recent study evaluating an autistic-led support programme for autistic adults, which found that in exploring their experiences as autistic individuals, participants developed a sense of unity in their diverse experiences of autism (Crane et al. 2020). Cresswell and Cage (2019) found that autistic participants who were more aligned with non-autistic culture generated more positive self-statements, which conflicts with our finding that autistic people who generated more positive autism attributes also had more positive collective self-esteem and stronger autism social identity. These discrepancies are likely due to the different constructs measured, with Cresswell and Cage’s study focussing on general self-statements and not autism related traits. Furthermore, they recruited adolescents, who may have a more negative view of autism than adults, as we found in our focus groups. The qualitative finding that individuals tended to find more coping strategies with time and came to a place of finding pride in their autism diagnosis, may well contribute to this shift in focus from autism-related challenges to strengths which happened over time. Furthermore, the adult participants had much later diagnosis than the younger participants, and potentially therefore felt a stronger need to identify with the diagnosis. It is probable that the older group would have struggled with autism-related challenges for many years without a full understanding of the reasons for this, and this may well have impacted on the centrality of their autism identity, once discovered.

### Strengths and Limitations of the Study

This study had a number of strengths. First, by using online recruitment methods we had a relatively large number of participants allowing us to account for the considerable heterogeneity of presentations and experiences of autistic people. In our focus groups, we gained a diverse range of opinions by including younger people currently in education and adults living in the community. Second, we conducted two separate studies and integrative analyses. This allowed us to test a mediation model without the complication of common method variance, which is a limitation of cross-sectional surveys. This meant that we were able to capture the relationship between one group of autistic individuals’ perceptions of autism and the social identification of other autistic participants—a valid test of the impact of the opinions of members of the wider community on individuals. The autism attributes were a novel intragroup predictor of autism social identification. In the mediation analysis, we were able to test the extent to which other in-group members' perceptions of the positivity of autism attributes affected participants' in-group identification. This eliminated the possibility that we should have tested an alternative mediation model, one in which Autism identification predicted the extent to which Autism attributes were perceived as positive.

A limitation of this study was that some of the survey participants reported not having received a diagnosis of autism. For the purpose of this study, an autism identity was more important than an autism diagnosis, due to the focus on sense of affiliation with the autistic community. When conducting the quantitative analysis, we divided the participants into those who were diagnosed with autism and those who were not. We did not find any differences between the two groups. It could be argued that individuals who self-select to take part in autism research are more likely to see autism as an important part of their identity by virtue of being aware of and responding to adverts directed at the autism community, which could affect the results. Furthermore, the average age of autism diagnosis for participants in our study was 29 years. Individuals who are diagnosed with autism later in life may construct their identities differently to those who are diagnosed in childhood. Future research could recruit individuals who are less focused on their autism identity, although this would be a challenging group to recruit, as well as those who received a diagnosis earlier in life. This study also had a focus on social identity processes in autistic individuals, and so did not take into account other factors that may contribute to collective self-esteem such as individual self-esteem or psychological well-being.

### Practice Implications

Our findings provide evidence that developing a balanced view of autism, with emphasis on the strengths associated with autism can have a positive effect for the collective self-esteem to the extent that the individual has a sense of affiliation with other autistic people. While this may happen ‘naturally’ for individuals over time, efforts by educational settings and support services may not only speed this process up, but also reduce the length of time spent living with a solely negative view of the condition. This may be particularly pertinent for early intervention with younger people post-diagnosis and our findings are highly supportive of endeavours such as the PEGASUS study, where developing a more comprehensive and balanced view of the autism diagnosis was the outcome following a group psychoeducational intervention for adolescents (Gordon et al. 2015). The findings also support the development of such groups for newly diagnosed adults, particularly when led by autistic individuals, as described by Crane et al. (2020). Such interventions can be delivered in educational or clinical settings following diagnostic assessment.

Online or community groups can foster a sense of positive autism identity (e.g., Brownlow and O'Dell 2006) and are likely to be beneficial for many autistic people. Moreover, given the links between social identification and mental health (Haslam et al. 2012), and the isolation that autistic individuals can struggle with (e.g., Billstedt et al. 2011), developing such groups could have a positive impact on the psychological wellbeing of autistic people. Beyond support for the autistic community, a wider societal awareness of the strengths of autism would benefit the autism community so that the burden is not placed solely on autistic individuals to raise awareness about the benefits of autism.

It is important to note that participants in this study spoke more of challenges than of strengths, and that the reality of autism for many individuals involves struggles and hardships. Many of these struggles were articulated as emotional difficulties such as disabling anxiety and depression and seen by people as integral to their autism identity. Assessment of emotional difficulties is often situated within the framework of ‘co-occurring’ or ‘co-morbid’ condition. While we are not arguing for a revision of the diagnostic criteria, it is important to note such distressing emotional difficulties are an important part of the autism experience and for which appropriately tailored, evidence-based support can be made available.

In sum, this study investigated the attributes of autism as identified by autistic adults, and we found that having a more positive view of autism—as determined by other members of the autistic community—was associated with a stronger sense of affiliation to the autistic community, and more positive autism collective self-esteem. These findings suggest that the autistic community benefits from a focus on strengths and positives associated with the condition, and that this strengths focus is something that older people are more likely to do than younger people.

## Footnotes

1 We conducted a one-way MANOVA with the independent variable ‘group’ (Autism diagnosed versus Autism self-identified), and the dependent variables of autism identification, collective self-esteem and valence of autism attributes. No significant group differences were found in autism identification (*F*(1,139) = 0.99, *p* = 0.32), collective self-esteem (*F*(1,139) = 0.98, *p* = 0.33), nor valence of autism attributes (*F*(1,138) = 0.03, *p* = 0.86). We further completed a one-way MANOVA with the independent variable ‘gender’, and the dependent variables of autism identification, collective self-esteem and valence of autism attributes. There was a significant effect of gender on autism social identity (*F*(2,132) = 4.07, *p* < 0.05), but not on collective self-esteem (*F*(1,132) = 0.14, *p* = 0.87), nor valence of autism attributes (*F*(2,131) = 1.40, *p* = 0.25). We looked at correlations between age and age of diagnosis and valence of autism attributes, autism identification and collective self-esteem. There was no significant correlation between age of diagnosis and valence of autism attributes (*r*(99) = .09, *p* = .37), nor between age of diagnosis and autism identification (*r*(99) = .05, *p* = .60), nor age of diagnosis and collective self-esteem (*r*(99) = .03, *p* = .77). There was no significant correlation between age and valence of autism attributes (*r*(135) = .08, *p* = .34) age and autism identification (*r*(136) = − .02, *p* = .86), nor age and collective self-esteem (*r*(136) = .02, *p* = .86).
